# Intravenous angiotensin II for the treatment of high-output shock (ATHOS trial): a pilot study

**DOI:** 10.1186/s13054-014-0534-9

**Published:** 2014-10-06

**Authors:** Lakhmir S Chawla, Laurence Busse, Ermira Brasha-Mitchell, Danielle Davison, Jacqueline Honiq, Ziyad Alotaibi, Michael G Seneff

**Affiliations:** Division of Intensive Care Medicine and Division of Nephrology, Department of Medicine, Veterans Affairs Medical Center, 50 Irving Street, NW, Washington, DC USA; Section of Critical Care Medicine, Department of Medicine, Inova Fairfax Medical Center, Falls Church, VA USA; Department of Anesthesiology and Critical Care Medicine, George Washington Medical Center, Washington, DC USA; Prince Sultan Medical Military City, Critical Care and Emergency Medicine, Riyadh, Saudi Arabia

## Abstract

**Introduction:**

Patients with distributive shock who require high dose vasopressors have a high mortality. Angiotensin II (ATII) may prove useful in patients who remain hypotensive despite catecholamine and vasopressin therapy. The appropriate dose of parenteral angiotensin II for shock is unknown.

**Methods:**

In total, 20 patients with distributive shock and a cardiovascular Sequential Organ Failure Assessment score of 4 were randomized to either ATII infusion (N =10) or placebo (N =10) plus standard of care. ATII was started at a dose of 20 ng/kg/min, and titrated for a goal of maintaining a mean arterial pressure (MAP) of 65 mmHg. The infusion (either ATII or placebo) was continued for 6 hours then titrated off. The primary endpoint was the effect of ATII on the standing dose of norepinephrine required to maintain a MAP of 65 mmHg.

**Results:**

ATII resulted in marked reduction in norepinephrine dosing in all patients. The mean hour 1 norepinephrine dose for the placebo cohort was 27.6 ± 29.3 mcg/min versus 7.4 ± 12.4 mcg/min for the ATII cohort (*P* =0.06). The most common adverse event attributable to ATII was hypertension, which occurred in 20% of patients receiving ATII. 30-day mortality for the ATII cohort and the placebo cohort was similar (50% versus 60%, *P* =1.00).

**Conclusion:**

Angiotensin II is an effective rescue vasopressor agent in patients with distributive shock requiring multiple vasopressors. The initial dose range of ATII that appears to be appropriate for patients with distributive shock is 2 to 10 ng/kg/min.

**Trial registration:**

Clinicaltrials.gov NCT01393782. Registered 12 July 2011.

## Introduction

Critically ill patients with shock requiring vasopressors are at a high risk of death. Distributive shock is the most common form, and is often caused by sepsis [1]. When shock is treated with vasopressors, two main classes of vasopressors are in the intensivists’ armamentarium: catecholamines and vasopressin-type peptides [1]. Currently, no specific type of vasopressor (for example, norepinephrine, vasopressin, dopamine) compared to another vasopressor has been shown to improve outcome [2]. All vasopressors have limitations and potential side effects. Patients treated with catecholamines for shock often develop tachyphylaxis, thereby limiting the utility of these agents, and high doses of catecholamines can cause direct cardiotoxicity [3]. The toxic potential of catecholamines has been recently demonstrated in a randomized clinical trial of septic shock patients treated with norepinephrine [4]. In this study, beta blockade with esmolol was shown to improve survival in these patients by decreasing the heart rate. Thus, vasopressors that are not inotropes or chronotropes may be useful in patients with shock. One such vasopressor is vasopressin, which is most commonly used as an adjuvant with catecholamines. Vasopressin has been shown to improve outcomes in patients with less severe septic shock, but has toxicity (that is, cardiac and mesenteric ischemia) at high doses and interacts with hydrocortisone [5]. In the subset of critically ill patients in whom mean arterial pressure (MAP) cannot be maintained with vasopressors, distributive shock is uniformly fatal. The addition of a rescue vasopressor in this setting could be useful.

Angiotensin II (ATII) is a naturally occurring hormone with endocrine, autocrine, paracrine, and intracrine hormonal effects. It is a potent direct vasoconstrictor, constricting both arteries and veins and increasing blood pressure [6]. It has a half-life in circulation of approximately 30 seconds, but while in tissue, its half-life may be as long as 15 to 30 minutes. Importantly, ATII increases secretion of antidiuretic hormone (ADH) and adrenocorticotropin hormone (ACTH), and may potentiate sympathetic effects by direct action on postganglionic sympathetic fibers. It also acts on the adrenal cortex, causing it to release aldosterone [6,7]. We hypothesized that ATII might serve a role as a useful vasopressor in the treatment of shock, but the appropriate dose of intravenous ATII to increase blood pressure is unknown. The dose of intravenous ATII previously described has varied across studies, but ranges from 0.4 ng/kg/min to as much as 40 ng/kg/min. The highest doses were reported in the cases of profound hypotension due to angiotensin-converting-enzyme inhibitor (ACEi) overdose [8,9]. We set out to determine the appropriate dose of ATII in the treatment of high output shock.

## Methods

The study was conducted at the George Washington University Hospital Intensive Care Unit, Washington DC, USA. The trial was registered on clinicatrials.gov (NCT01393782) and the study protocol was approved by the Food and Drug Administration (IND# BB-IND-11592). The protocol was approved by the George Washington University Institutional Review Board. Written informed consent was obtained from each participating patient or appropriate surrogate prior to enrollment. The investigators performed all experimental procedures and the study coordinators recorded the data.

### Study patients

Patients were eligible for randomization if they were older than 21 years and were deemed to have high-output shock, which was defined as a cardiovascular sequential organ function assessment (SOFA) score of 4 as well as a cardiac index >2.4 L/min/BSA 1.73 m^2^ [10]. Patients were also required to have an indwelling arterial line and urinary catheter as part of standard care and expected to be present for at least 12 hours during the study intervention. In addition, the treating team had to deem the subject adequately volume-resuscitated and clinically assessed not to be volume-responsive (that is, a fluid bolus would fail to increase cardiac index by 15%). Standard of care at our institution is to resuscitate with 20 to 30 cc/kg of crystalloid as initial resuscitation. Exclusion criteria included patients with acute coronary syndrome, a known history of vasospasm or asthma, any patients currently experiencing bronchospasm, patients with active bleeding with an anticipated need for transfusion of >4 units of packed red blood cells, hemoglobin <7 g/dL, or any other condition that would contraindicate drawing serial blood samples.

### Treatment assignments

Upon enrollment in the study, patients were randomly assigned following simple randomization procedures (computerized random numbers) to receive either ATII acetate infusion (Clinalfa, Bachem AG, Hauptstrasse 144, 4416 Bubendorf, Switzerland) or a placebo infusion (hereafter referred to as the study drug and placebo, respectively). Randomization was accomplished by the Investigational Drug Services (IDS) at George Washington University Hospital. For the duration of the entire study, only the IDS was aware of each patient’s treatment assignment. Unblinding was done after all 20 patients were enrolled. All other clinical personnel, including the investigators, clinical support staff, the patients and their families were unaware of the treatment assignment for the duration of the study.

### Drug infusion

**Figure 1:**
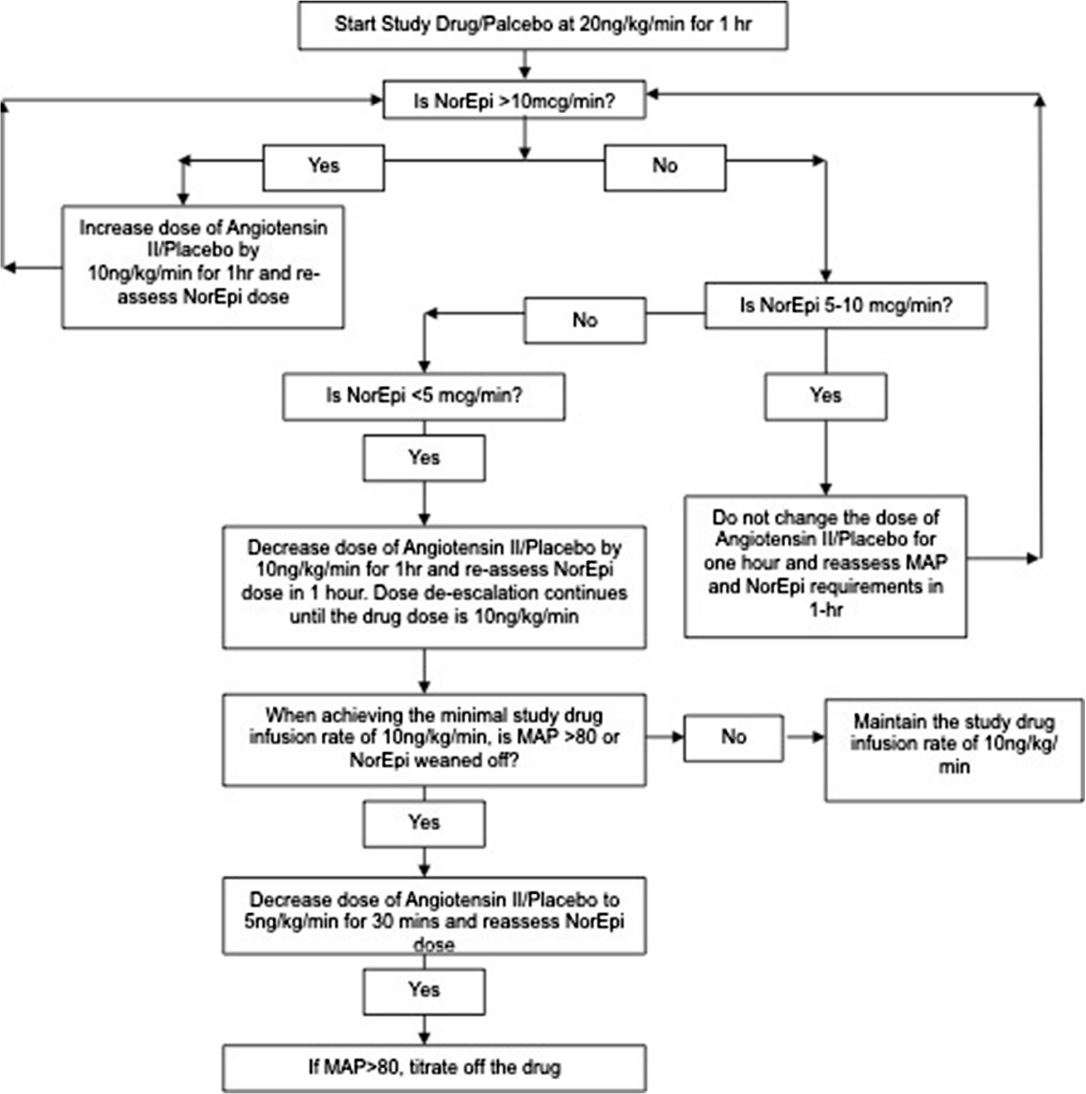
**Study drug titration protocol.** NorEpi, norepinephrine; MAP, mean arterial pressure.

Enrolled patients were randomized to receive the study drug infusion in normal saline calculated to run at a drip rate corresponding to an initial concentration of 20 ng/kg/min, plus the standard-of-care treatment for high-output shock. The study drug was prepared in an opaque cellophane bag, the contents of which were unknown to the investigators, nurses or anyone else taking direct care of the patient. The study drug was administered for a total of 6 hours, with dose (and corresponding drip rate) adjustments made hourly. Study drug dose adjustments were determined per a pre-specified protocol, based on the concomitant requirements of standard-of-care therapy (in all cases, norepinephrine infusion plus vasopressin, epinephrine and/or phenylephrine infusions) needed to maintain a MAP at or above 65 mm Hg, which is the standard practice at our institution. The study drug titration protocol was designed to elucidate the dose of ATII that was required (in conjunction with a norepinephrine dose between 5 and 10 mcg/min) to achieve the aforementioned standard MAP goal of 65 mm Hg. The dose titration protocol is shown on Figure 1. The maximum allowable dose for the ATII titration was 40 ng/kg/min, and the minimum was 5 ng/kg/min. At the end of 6 hours, the study drug infusion was titrated off by halving it every 10 minutes until the study drug infusion dose was below 5 ng/kg/min, after which it was discontinued.

### Endpoints

The primary endpoint was the effect of the ATII infusion on the standing dose of norepinephrine that was required to maintain a MAP of 65 mmHg. The secondary endpoints included the effect of the ATII infusion on urine output, serum lactate, cardiac output, and 30-day mortality.

### Statistical analysis

This was a safety and dose-finding feasibility study. We analyzed a small cohort of patients, consistent with similar studies of this nature. We estimated that a population of 20 patients, 10 patients in each arm, would generate a basis for determining if there was sufficient signal for ATII to affect the dose of norepinephrine at the doses outlined herein. An independent data and safety monitor (DSM) was assigned and reviewed all adverse events. The DSM had the power to be unblinded and halt the study at any time during the conduct of the study.

We assessed the distribution of demographic and clinical variables. Difference between proportions of patients with certain variables was assessed with the chi-square, Fisher exact, Student *t*-, or Mann–Whitney test as appropriate. The primary endpoint of the effect of the study drug infusion on the standing dose of norepinephrine was calculated using a general estimating equation analysis and is presented as the mean dose of norepinephrine (mcg/min) and study drug infusion (in ng/kg/min) at hourly intervals.

A generalized estimating equation was used to model the response to the study drug over time, with standard-of-care vasopressor hourly readings beginning at 1 hour prior to, through 8 hours after the initiation of the study drug, using the SAS Genmod procedure (version 9.3, Cary, NC, USA). Correlation structure was defined as auto-regressive to account for the likely higher correlation between time points that were closer together. In this model, the main effect of drug examines the mean response to each drug averaged across times. The main effect of time examines the mean response at each time point averaged across drugs, and the drug multiplied by time interaction examines whether the change over time differs between drugs. All values are reported as mean ± SD unless otherwise specified. All other statistical analysis was completed using SPSS 18, Chicago, IL, USA.

## Results

**Figure 2:**
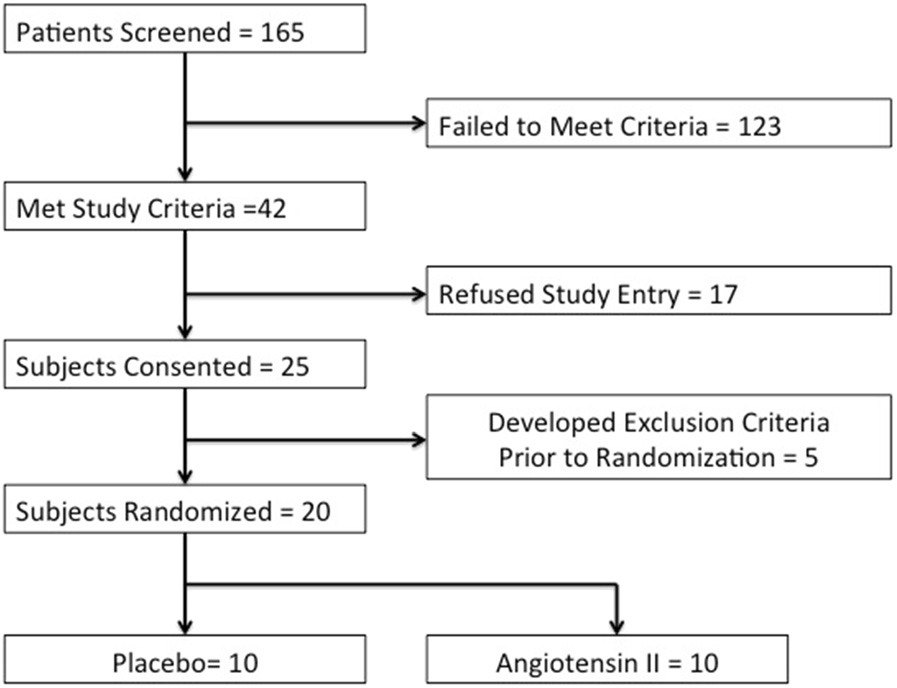
**Patient flow diagram.**

The flow of patients into the study is reported in Figure 2: 20 patients underwent randomization and all 20 patients were enrolled in and completed the study (Figure 1). Baseline characteristics of the two groups are shown in Table 1. The mean age for all study subjects was 62.9 ± 15.8 years. Of the patients, 75% were male, 45% were Caucasian and 40% were African American. Baseline SOFA and acute physiology and chronic health evaluation II (APACHE II) scores were 15.9 ± 3.0 and 30.6 ± 8.9, respectively. Of the 20 patients 19 were receiving concomitant vasopressin at a dose of 0.02 to 0.08 u/min. Vasopressin doses were not adjusted during the study period.

**Table 1:** **Baseline demographic and clinical data**

	**Full cohort**	**SD**	**ATII**	**SD**	**Placebo**	**SD**	***P*** **-value** ^**1**^
Age, years	62.85	15.81	68.40	17.46	57.30	12.44	0.12
Male, number	15	NA	6	NA	9	NA	0.30
Race, number							
Caucasian	9	NA	6	NA	3	NA	0.37
Black	8	NA	3	NA	5	NA	0.65
Other	3	NA	1	NA	2	NA	1.00
Severity of illness		NA		NA		NA	
Baseline SOFA score	15.90	2.97	14.9	2.81	16.90	2.92	0.14
APACHE score	30.60	8.86	27.2	9.67	34.00	6.83	0.09
Past medical history, number							
IHD	2	NA	1	NA	1	NA	1.00
CHF	2	NA	2	NA	0	NA	0.47
COPD	2	NA	2	NA	0	NA	0.47
DM	7	NA	4	NA	3	NA	1.00
CKD	7	NA	3	NA	4	NA	1.00
HD	1	NA	0	NA	1	NA	1.00
Liver disease	9	NA	5	NA	4	NA	1.00
Cancer	6	NA	1	NA	5	NA	0.14
IS	6	NA	1	NA	5	NA	0.14
Steroids	3	NA	1	NA	2	NA	1.00
Hypertension	9	NA	4	NA	5	NA	1.00
CVA	5	NA	4	NA	1	NA	0.30
AKI	17	NA	9	NA	8	NA	1.00
Laboratory measurements mean							
White blood cells	17.38	NA	19.0	16.0	15.72	12.3	0.61
Hgb	9.45	NA	9.16	2.14	9.73	2.45	0.59
Creatinine	2.33	NA	1.89	1.03	2.76	1.34	0.12
pH	7.33	NA	7.34	0.11	7.32	0.12	0.63
Lactate	5.83	NA	4.59	3.11	7.06	5.16	0.21
Baseline vasopressor doses^2^							
Norepinephrine	25.05	17.03	19.80	11.67	30.30	20.37	0.18
Vasopressin	0.04	0.02	0.03	0.02	0.05	0.02	0.10

Results are presented as mean and SD or number. ^1^ *P*-values for continuous variables calculated using Student's *t*-test. *P*-values for discrete variables calculated using Fisher exact test. ^2^One patient in the placebo group received phenylephrine infusion prior to initiation of angiotensin II (ATII) versus no patients in the ATII group. One patient in the placebo group received epinephrine versus no patients in the ATII group. SOFA, sequential organ function assessment; APACHE, acute physiology and chronic health evaluation II; IHD, ischemic heart disease; CHF, congestive heart failure; COPD, chronic obstructive pulmonary disease; DM, diabetes mellitus; CKD, chronic kidney disease; HD, hemodialysis; IS, immunocompromised state; CVA, cerebrovascular accident; AKI, acute kidney injury; Hgb, hemoglobin; NA, to represent not analysed, not applicable, or not available.

**Figure 3:**
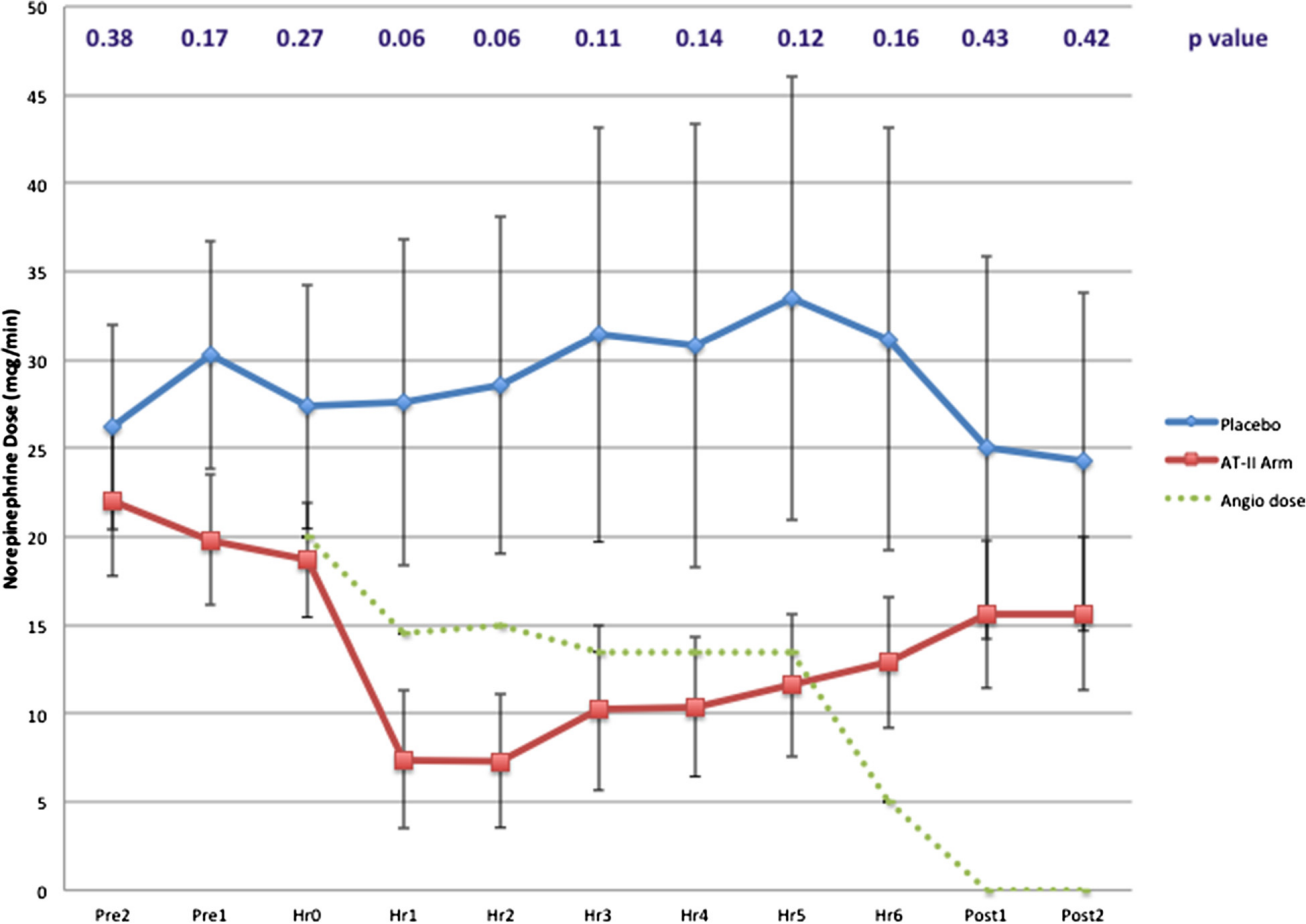
**Changes in norepinephrine dose with concurrent angiotensin II.** AT-II arm, angiotensin-II arm; Angio dose, angiotensin-II dose.

ATII resulted in a reduction in norepinephrine dosing in all patients (Figure 3). The mean hour-1 norepinephrine dose for the placebo cohort was 27.6 ± 29.3 mcg/min versus 7.4 ± 12.4 mcg/min for the ATII cohort (*P* = 0.06). Hour 2 norepinephrine dosing for the placebo cohort was 28.6 ± 30.2 mcg/min versus 7.3 ± 11.9 mcg/min in the ATII cohort (*P* = 0.06). Throughout the study period, the mean ATII dose was reduced from 20 ng/kg/min at hour 0 to 5 ng/kg/min at hour 6 before being titrated off by hour 7 (one hour post-infusion). Despite this down-titration of ATII, norepinephrine doses remained substantially lower in the ATII cohort than the placebo cohort, though the effect approached statistical significance only at hours 1 and 2. Upon cessation of the ATII infusion, mean norepinephrine rebounded concomitantly.

Using a general estimating equation model with time defined as a continuous variable, in order to obtain a global test of interaction effect, the main effect of treatment (study drug versus placebo) was not significant (*P* = 0.13), nor was the effect of time (*P* =0.30), nor was the treatment multiplied by time interaction (*P* = 0.76). When time was defined as a class variable with hour-1 defined as the reference group, in order to examine specific time points, the drug effect (*P* = 0.14) and time effect (*P* = 0.18 at time 0, *P* = 0.51 at time 1) both remained non-significant. The product of drug multiplied by time interaction showed a trend level of significance at 1 hour and 2 hours (*P* = 0.06).

Adverse events most commonly experienced by all patients were metabolic disorders with alkalosis occurring in four patients in the ATII group and no patients in the placebo group (*P* = 0.09). The most common adverse event thought to be attributable to ATII was hypertension, which occurred in 20% of patients receiving ATII (*P* = 0.58). In both of these patients, the study drug infusion was stopped, per protocol, in order to achieve MAP goals. Table 2 lists adverse events.

**Table 2:** **Adverse events**

**Organ system**	**Total**	**ATII**	**Placebo**	***P*** **-value**
Metabolic disorders	16	11	5	
Acidosis		2	3	1.00
Alkalosis		4	0	0.09
Blood or lymphatic disorders	7	3	4	1.00
Respiratory disorders	6	3	3	1.00
Worsening respiratory failure		1	3	0.58
Wheezing		1	0	1.00
Cardiac disorders	12	7	5	0.65
Hypertension^1^		2	0	0.58
Hypotension		2	1	1.00
Atrial fibrillation		2	0	0.47
Renal disorders^2^	7	6	2	0.17
Decreased urine output		3	1	0.58
Worsening acute kidney injury		0	2	0.47
Other disorders^3^	8	5	3	0.65
Worsening multiple organ system failure		2	3	1.00

The results were presented as number of adverse events. ^1^Angiotensin II (ATII) infusion was discontinued in two patients due to hypertension. ^2^Of 20 patients 17 exhibited pre-existing acute kidney injury (AKI), including 8 patients receiving placebo and 9 patients receiving ATII. Of the three patients who did not have pre-existing AKI, one patient developed AKI and received ATII. ^3^Includes worsening multiple organ system failure, fever, lower extremity edema, and thigh hematoma. *P*-values were calculated using the Fisher exact test.

Urine output, cardiac output, central venous pressure, and MAP are shown in Table 3. The 30-day mortality for the two groups was similar for the ATII cohort and the placebo cohort (50% versus 60%, *P* = 1.00).

**Table 3:** **Secondary outcomes**

	**Hour −2**	**Hour −1**	**Hour 0**	**Hour 1**	**Hour 2**	**Hour 3**	**Hour 4**	**Hour 5**	**Hour 6**	**Hour 7**	**Hour 8**
**Urine output, cc**											
ATII	41.7 (51.7)	28.6 (32.4)	45.9 (96.5)	31.1 (58.0)	33.7 (67.1)	42.6 (59.8)	35.9 (50.0)	34.4 (57.2)	36.1 (38.3)	27.2 (33.3)	23.8 (27.3)
Placebo	29.5 (69.8)	12.4 (23.2)	23.5 (41.8)	17.5 (25.7)	17.0 (32.0)	16.3 (24.6)	17.0 (34.7)	16.8 (30.4)	14.8 (26.3)	18.0 (27.3)	23.0 (34.4)
**Cardiac output, L/min**											
ATII	7.0 (2.7)	6.0 (3.1)	6.6 (2.6)	6.3 (2.5)	6.2 (2.5)	5.9 (2.7)	6.5 (2.4)	6.1 (2.6)	6.7 (3.3)	6.3 (2.9)	7.5 (3.1)
Placebo	6.3 (1.2)	6.9 (2.5)	6.5 (1.7)	6.9 (1.8)	6.4 (1.7)	6.8 (3.0)	7.3 (2.2)	6.8 (1.5)	7.3 (1.8)	6.9 (2.5)	7.0 (2.3)
**Central venous pressure**											
ATII	12.7 (5.5)	12.9 (7.0)	14.1 (8.9)	14.8 (7.3)	14.6 (7.4)	14.8 (8.8)	11.7 (3.9)	12.1 (4.8)	10.0 (2.9)	12.6 (4.8)	11.8 (3.8)
Placebo	16.0 (3.0)	9.7 (2.1)	12.6 (7.8)	15.7 (9.3)	17.3 (9.0)	15.7 (7.3)	16.4 (8.6)	16.3 (6.7)	14.2 (6.5)	14.4 (7.1)	13.2 (5.6)
**Mean arterial pressure**											
ATII	71.2 (13.6)	72.3 (11.2)	68.8 (7.0)	74.8 (8.4)	69.8 (8.6)	73.1 (12.5)	75.3 (14.2)	68.9 (8.1)	73.0 (10.5)	72.3 (11.9)	73.6 (11.5)
Placebo	71.2 (9.2)	71.8 (6.5)	73.0 (12.6)	72.8 (9.5)	67.8 (6.6)	70.1 (6.4)	71.3 (7.8)	73.0 (4.7)	75.9 (9.4)	74.0 (10.6)	74.5 (13.2)
**Lactate**											
ATII	NA	NA	4.6 (3.1)	NA	NA	NA	NA	NA	5.2 (4.1)	NA	NA
Placebo	NA	NA	7.1 (5.2)	NA	NA	NA	NA	NA	5.7 (3.9)	NA	NA

All variables are presented as mean (SD). ATII, angiotensin II; NA, to represent not analysed, not applicable, or not available.

## Discussion

We report the findings of the first prospective randomized placebo-controlled trial of ATII in the treatment of high-output shock. Our efforts were intended as a proof-of-concept and dose-finding study, as well as an attempt to generate hypotheses in advance of larger future studies. To this end, we have shown that ATII can be an effective pressor agent at a dose range of 5 to 40 ng/kg/min. More specifically, we believe that a starting dose of 2 to 10 ng/kg/min may be an appropriate starting dose in the treatment of high-output shock when used in conjunction with standard-of-care vasopressors.

ATII has been used previously for the treatment of hypotension in a handful of cases. Newby *et al*. describe the successful treatment of a patient with an ACEi overdose and profound shock, using an ATII infusion [11]. Multiple case reports have shown potential utility for ATII in the treatment of septic shock with catecholamine infusions [12–16].

While all patients in the present study had a response to the ATII infusion, we observed significant heterogeneity. Of the ten patients who received ATII, two had a modest response, while two were exquisitely sensitive to ATII, which was an unexpected finding. In the two highly sensitive patients, the norepinephrine infusion was titrated off per protocol, and the ATII dose was at its lowest allowable dose of 5 ng/kg/min and the patients remained hypertensive with MAP of >90 mm Hg, despite norepinephrine being titrated off. As hypertension is not part of our standard of care, the investigators halted the infusion, and the ATII was weaned off. In both cases the need for norepinephrine was rapidly re-established. Pre-clinical studies demonstrate that animals that become septic after pre-treatment with enalapril are resistant to norepinephrine as a vasopressor [17]. Based on these findings, we hypothesized that the two patients who were very sensitive to ATII may have been receiving an angiotensin-converting enzyme inhibitor prior to developing septic shock, thus, explaining the exquisite sensitivity to ATII infusion. However, a detailed chart review revealed incomplete information, and we were unable to document pre-morbid exposure to angiotensin-converting enzyme inhibitor. Therefore, the ATII sensitivity that we observed could be due to other mechanisms that have not been elucidated.

We observed that ATII may have synergy with other vasopressors (that is, catecholamines and vasopressin), but may also have another important indication in critically ill patients. Previous work in pre-clinical studies suggests that septic animals suffer acute kidney injury in part due to intra-glomerular hypotension induced by efferent arteriole vasodilation. In these models, intravenous infusion with ATII restores creatinine clearance and urine output [18]. While the present study was underpowered to elucidate any effect on urine output, we expect further large-scale studies will clarify the effect of ATII on kidney function in high-output shock. In addition, ATII is a potent vasopressor without inotropic or chronotropic properties [19]. Recent randomized controlled trials in patients with septic shock treated with norepinephrine suggest that less chronotropy may be desirable and may lead to a survival benefit [4]. Based on these findings, for patients who require norepinephrine and are tachycardic, ATII may be particularly useful. We hypothesize that for patients with severe hypotension, lower doses of multiple vasopressors with differing mechanisms of action may be more efficacious and less toxic than high doses of one type of vasopressor (that is, catecholamines).

The study had multiple strengths. First, the study was a randomized double-blind controlled trial with an appropriate placebo-control arm. Second, it was of pragmatic design, as it was the intent of the investigators to enroll patients receiving standard-of-care treatment for high-output shock. As such, all patients had received a priori appropriate monitoring and therapeutic interventions (including central venous lines, bladder catheters, arterial lines, and cardiac output monitoring devices). There was no additional need for any specialized equipment of procedures prior to enrollment in the study. Third, all enrolled patients had a documented need for high-dose vasopressor therapy despite volume therapy, as evidenced by the cardiac index entry criteria. This, we believe, is in keeping with the current practice of addressing volume responsiveness in a hypotensive patient prior to initiation of vasopressor therapy. Finally, as part of our study protocol, we employed the use of a data safety monitor, who had the ability to unblind data and evaluate for adverse events as well as halt the study, neither of which occurred.

The study had several limitations. First, the study had a modest sample size. As such, we were unable to make conclusions about the effect of ATII on urine output due to the high incidence of oliguria and renal replacement therapy in both the ATII group and the placebo group. Moreover, this study was not powered adequately to discern a difference in mortality between the ATII and placebo groups. Second, there were some imbalances between our placebo arm and our drug arm, the former of which were younger, but sicker (according to both SOFA and APACHE II scores). It is possible that differences in these two populations influenced the effectiveness of ATII. Finally, inclusion criteria for enrollment in the study were such that the resulting study population was critically ill, with an expected mortality in excess of 50%. Indeed, our requirement of a cardiovascular SOFA score of 4 (indicating a norepinephrine dose of 0.1 mcg/kg/min) would, on average, equate to a minimum norepinephrine dose of 9.3 mcg/min, which based on our study drug titration protocol (Figure 1), would leave little room for titration. In order to allow for the possibility of a meaningful signal upon initiation of ATII, we preferentially considered patients with a substantially higher starting dose of norepinephrine (20 to 30 mcg/min, as evidenced in Figure 3). Moreover, we preferentially considered patients with an upward-trending norepinephrine requirement, signifying refractoriness to therapy. Based on these facts, our results may not be generalizable to a less sick population. However, we foresee a use for ATII in a significant critically ill population, for whom multiple vasopressors are required.

## Conclusions

The initiation of an ATII infusion in patients receiving norepinephrine for septic shock resulted in a marked decrease in norepinephrine doses. ATII may be effective as a novel pressor agent in the treatment of high-output shock. Initial dosing ranges are most likely between 2 and 10 ng/kg/min. In our pilot study, the drug appears to be well-tolerated. Further randomized placebo-controlled trials to more fully elucidate the role of ATII as a vasopressor in the treatment of shock are warranted.

## Key messages

- Angiotensin II improved blood pressure in patients with high-output shock and multiple vasopressors.
- Angiotensin II may have a role as a rescue vasopressor.
- The response to angiotensin II in a modest-sized study were highly variable.
- The precise role of angiotensin II in the treatment of hypotension and shock needs further study.
- Angiotensin II was well-tolerated in this pilot study.

## Abbreviations

ACE: angiotensin-converting-enzyme
ACEi: angiotensin-converting-enzyme inhibitor
ACTH: adrenocorticotropin hormone
ADH: antidiuretic hormone
AKI: acute kidney injury
APACHE: acute physiology and chronic health evaluation II
ATII: angiotensin II
CHF: congestive heart failure
CKD: chronic kidney disease
COPD: chronic obstructive pulmonary disease
CVA: cerebrovascular accident
CVP: central venous pressure
DM: diabetes mellitus
DSM: data and safety monitor
HD: hemodialysis
Hgb: hemoglobin
IDS: Investigational Drug Services
IHD: ischemic heart disease
MAP: mean arterial pressure
MOSF: multiple organ system failure
SOFA: sequential organ function assessment
WBC: white blood cell

## References

[1] Vincent JL, De Backer D (2013). Circulatory shock. N Engl J Med.

[2] Myburgh JA, Higgins A, Jovanovska A, Lipman J, Ramakrishnan N, Santamaria J, CAT Study investigators (2008). A comparison of epinephrine and norepinephrine in critically ill patients. Intensive Care Med.

[3] Rona G (1985). Catecholamine cardiotoxicity. J Mol Cell Cardiol.

[4] Morelli A, Ertmer C, Westphal M, Rehberg S, Kampmeier T, Ligges S, Orecchioni A, D'Egidio A, D'Ippoliti F, Raffone C, Venditti M, Guarracino F, Girardis M, Tritapepe L, Pietropaoli P, Mebazaa A, Singer M (2013). Effect of heart rate control with esmolol on hemodynamic and clinical outcomes in patients with septic shock: a randomized clinical trial. JAMA.

[5] Russell JA, Walley KR, Singer J, Gordon AC, Hebert PC, Cooper DJ, Holmes CL, Mehta S, Granton JT, Storms MM, Cook DJ, Presneill JJ, Ayers D, VASST Investigators (2008). Vasopressin versus norepinephrine infusion in patients with septic shock. N Engl J Med.

[6] Basso N, Terragno NA (2001). History about the discovery of the renin-angiotensin system. Hypertension.

[7] Struthers AD, MacDonald TM (2004). Review of aldosterone- and angiotensin II-induced target organ damage and prevention. Cardiovasc Res.

[8] Jackson T, Corke C, Agar J (1993). Enalapril overdose treated with angiotensin infusion. Lancet.

[9] Trilli LE, Johnson KA (1994). Lisinopril overdose and management with intravenous angiotensin II. Ann Pharmacother.

[10] Vincent JL, Moreno R, Takala J, Willatts S, De Mendonca A, Bruining H, Reinhart CK, Suter PM, Thijs LG (1996). The SOFA (Sepsis-related Organ Failure Assessment) score to describe organ dysfunction/failure. On behalf of the Working Group on Sepsis-Related Problems of the European Society of Intensive Care Medicine. Intensive Care Med.

[11] Newby DE, Lee MR, Gray AJ, Boon NA (1995). Enalapril overdose and the corrective effect of intravenous angiotensin II. Br J Clin Pharmacol.

[12] Wray GM, Coakley JH (1995). Severe septic shock unresponsive to noradrenaline. Lancet.

[13] Whiteley SM, Dade JP (1996). Treatment of hypotension in septic shock. Lancet.

[14] Ryding J, Heslet L, Hartvig T, Jonsson V (1995). Reversal of ‘refractory septic shock’ by infusion of amrinone and angiotensin II in an anthracycline-treated patient. Chest.

[15] Thomas VL, Nielsen MS (1991). Administration of angiotensin II in refractory septic shock. Crit Care Med.

[16] Yunge M, Petros A (2000). Angiotensin for septic shock unresponsive to noradrenaline. Arch Dis Child.

[17] Correa TD, Jeger V, Pereira AJ, Takala J, Djafarzadeh S, Jakob SM (2014). Angiotensin II in Septic Shock: Effects on Tissue Perfusion, Organ Function, and Mitochondrial Respiration in a Porcine Model of Fecal Peritonitis. Crit Care Med.

[18] Wan L, Langenberg C, Bellomo R, May CN (2009). Angiotensin II in experimental hyperdynamic sepsis. Crit Care.

[19] Goldsmith SR, Hasking GJ (1991). Effect of a pressor infusion of angiotensin II on sympathetic activity and heart rate in normal humans. Circ Res.

